# Static and Dynamic Accuracy and Occlusion Robustness of SteamVR Tracking 2.0 in Multi-Base Station Setups

**DOI:** 10.3390/s23020725

**Published:** 2023-01-08

**Authors:** Lara Kuhlmann de Canaviri, Katharina Meiszl, Vana Hussein, Pegah Abbassi, Seyedeh Delaram Mirraziroudsari, Laurin Hake, Tobias Potthast, Fabian Ratert, Tessa Schulten, Marc Silberbach, Yannik Warnecke, Daniel Wiswede, Witold Schiprowski, Daniel Heß, Raphael Brüngel, Christoph M. Friedrich

**Affiliations:** 1 Department of Computer Science, University of Applied Sciences and Arts Dortmund (FH Dortmund), 44227 Dortmund, Germany; 2 Institut für die Digitalisierung von Arbeits- und Lebenswelten (IDiAL), University of Applied Sciences and Arts Dortmund (FH Dortmund), 44227 Dortmund, Germany; 3 Institute for Medical Informatics, Biometry and Epidemiology (IMIBE), University Hospital Essen, 45122 Essen, Germany; 4 Institute for Artificial Intelligence in Medicine (IKIM), University Hospital Essen, 45131 Essen, Germany

**Keywords:** SteamVR Tracking 2.0, SteamVR Base Station 2.0, VIVE Tracker (3.0), virtual reality, static tracking accuracy, occlusion robustness

## Abstract

The tracking of objects and person position, orientation, and movement is relevant for various medical use cases, e.g., practical training of medical staff or patient rehabilitation. However, these demand high tracking accuracy and occlusion robustness. Expensive professional tracking systems fulfill these demands, however, cost-efficient and potentially adequate alternatives can be found in the gaming industry, e.g., SteamVR Tracking. This work presents an evaluation of SteamVR Tracking in its latest version 2.0 in two experimental setups, involving two and four base stations. Tracking accuracy, both static and dynamic, and occlusion robustness are investigated using a VIVE Tracker (3.0). A dynamic analysis further compares three different velocities. An error evaluation is performed using a Universal Robots UR10 robotic arm as ground-truth system under nonlaboratory conditions. Results are presented using the Root Mean Square Error. For static experiments, tracking errors in the submillimeter and subdegree range are achieved by both setups. Dynamic experiments achieved errors in the submillimeter range as well, yet tracking accuracy suffers from increasing velocity. Four base stations enable generally higher accuracy and robustness, especially in the dynamic experiments. Both setups enable adequate accuracy for diverse medical use cases. However, use cases demanding very high accuracy should primarily rely on SteamVR Tracking 2.0 with four base stations.

## 1. Introduction

Virtual Reality (VR) allows users to enter immersive virtual spaces and thus become part of them, providing illusions of an alternative reality. Use cases for VR applications are rapidly increasing, primarily within the gaming and entertainment industry, and can go as far as the Metaverse [1]. In VR, active control of user body movements [2] or objects in the real world are reflected by means of sensors. Due to general technological advances within the last decade, VR applications have experienced a considerable upswing in research and healthcare, especially in the field of training simulations for medical procedures and rehabilitation [3]. In comparison with case- and expert-driven practical training of medical staff, training simulations represent a cost-efficient and high-available alternative for procedure practice. For example, sonography simulations may solely rely on exemplary case recordings and information from a transducer dummy enhanced with sensors, operated on a patient dummy [4]. When transferred to VR [5], tracking technology is used to capture positions, poses, and movements of involved objects, i.e., simulated counterparts of medical device and patient dummies. This provides the basis for an immersive simulation with precise conduction of training sessions [6,7,8,9,10,11,12,13]. In the field of telemedicine, VR applicaions are used for planning, as well as treating and rehabilitating patients [14,15,16]. In rehabilitation, tracking technology is used to capture and document patient body movement in real-time, and specialized VR-based training serves the purpose of improvement or recovery of patient motor functions [17,18,19]. However, in order to be applicable for clinical use cases, tracking systems must meet certain quality criteria, such as high positional and orientational accuracy, as well as low latency [20,21]. Therefore, evaluating these criteria for a system is essential before applications can rely on it.

Professional systems, primarily used in industry, offer high tracking accuracy but are costly. Basic configurations demand thousands of USD of investment and, depending on requirements, the price can scale up to hundreds of thousands of USD. Potential cost-efficient alternatives developed by the gaming industry are thus increasingly coming into focus. Initially developed for entertainment purposes, these systems may offer adequate tracking accuracy for specific types of professional use cases. One example for such a low-cost tracking system is SteamVR Tracking (SteamVR Tracking: https://partner.steamgames.com/vrlicensing (accessed on 20 December 2021)) developed by the Valve Corporation (Valve Corporation: https://www.valvesoftware.com/de/ (accessed on 9 March 2022)), comprising hardware and software components that enable real-time tracking. Initially released in 2016, its second version SteamVR Tracking 2.0 has been available since 2018. It operates on optimized hardware, the SteamVR Base Stations 2.0 (SteamVR Base Station 2.0: https://www.vive.com/de/accessory/base-station2/ (accessed on 18 February 2022)), which notably extends its capabilities. While the majority of research related to evaluation of SteamVR Tracking used its initial version, only few works have yet addressed SteamVR Tracking 2.0. An in-depth evaluation of certain aspects, e.g., a dynamic analysis or a multi-base station setup comparison, is currently not available.

The research at hand aims to provide such an in-depth evaluation of SteamVR Tracking 2.0 and its revised SteamVR Base Stations 2.0 for yet unaddressed questions. In order to fill gaps regarding the impact of amounts of involved base stations, a comparison of two setups is performed: one involving two, and the other involving four base stations. As part of the experiments, static tracking accuracy for position and orientation is examined for all axes, as well as dynamic tracking accuracy for translation during a spiral trajectory with three different velocities. Furthermore, robustness against the occlusion of base stations is analyzed, considering different positioning. A VIVE (VIVE: https://www.vive.com/ (accessed on 20 December 2022)) Tracker (3.0) (VIVE Tracker (3.0): https://www.vive.com/de/accessory/tracker3/ (accessed on 18 February 2022)) is used for measurements, for which a Universal Robots (Universal Robots: https://www.universal-robots.com/ (accessed on 9 March 2022)) UR10 robot arm (Universal Robots UR10: https://web.archive.org/web/20190112152822/, https://www.universal-robots.com/products/ur10-robot/ (accessed on 10 March 2022)) [22] serves as Ground Truth System (GTS). This continues prior work [4] that evaluated SteamVR Tracking 1.0 with two SteamVR Base Stations 1.0 for medical device tracking.

The paper is structured as follows: Section 2 provides a summarized overview of related work. Section 3 then elaborates used materials and methods, including SteamVR Tracking hardware and software, the experimental environment, and setups, as well as evaluation procedures. Section 4 reports experiment results, which are discussed in Section 5, pointing out limitations for future work. Conclusions are drawn in Section 6, highlighting the main findings and contributions.

## 2. Related Work

Table 1 provides a summarized overview of related work that is focused on the evaluation of SteamVR Tracking in terms of tracking performance. The overview uses a nomenclature for specific analysis types, described in the following in alphabetical order.

**Dynamic analysis**: A setting with dynamic positions of the tracked object during measurements, e.g., for baseline accuracy determination.
**Dynamic precision analysis**: A setting with a dynamic analysis during repeated measurement runs, e.g., for repeatability determination.
**Loss of tracking**: A state with the tracking system losing the connection to the tracked object.
**Orientational accuracy**: A characteristic describing the deviation of the measured positions to the real positions at the rotational movement.
**Partial occlusion**: A state where scan information is partially lost due to occlusion of at least one camera/scanner (depending on the tracking system).
**Positional accuracy**: A characteristic describing the deviation of the measured positions to the real positions at the translational movement.
**Reliability analysis**: A setting with static or dynamic positions of at least two trackers, e.g., for intra- and inter-tracker accuracy determination.
**Static analysis**: A setting with static positions of the tracked object during measurements, e.g., for baseline accuracy determination.
**Static precision analysis**: A setting with a static analysis during repeated measurement runs, e.g., for repeatability determination.
**System latency**: A characteristic indicating the time between physical movement of the tracked object and this movement being reflected on the screen.
**Time performance analysis**: A setting with static or dynamic positions of the tracked object during measurements, e.g., for system latency determination.
**Total occlusion**: A state where scan information is completely lost for a certain time due to occlusion of the tracked object.

The majority of related work is based on the SteamVR Tracking 1.0 and two first generation SteamVR Base Stations (1.0) [4,21,23,24,25,26,27,28]. In these studies, performance of SteamVR Tracking and sometimes other professional tracking systems were compared against a GTS. Other professional trackings were, e.g., OptiTrack (OptiTrack: https://optitrack.com/ (accessed on 10 March 2022)) [4,23], PhaseSpace (PhaseSpace: https://www.phasespace.com/ (accessed on 10 March 2022)) [24], and Vicon (Vicon: https://www.vicon.com/ (accessed on 10 March 2022)) [25,29]). Based on specific experimental setups, single or multiple analyses were performed to determine performance characteristics based on single or multiple measurement runs. Heterogeneous results were reported for the accuracy of SteamVR Tracking, and static positional errors of individual trials varied between ≤1 mm [4,23,26,28] and 5.8 mm [25]. When SteamVR Tracking 1.0 was compared against a professional tracking system, there were no major differences in measurement outcomes. While some also performed dynamic analyses in terms of performance [23,26,27,29], others investigated tracking behavior depending on temporary and complete occlusion or on different distances between base stations [21,28]. Reported dynamic analyses [23,26,27,29] show a considerable variation of errors ranging from the submillimeter range [26,27,29] up to several hundreds of mm [23,26], strongly depending on used velocities and trajectories. In addition, a low latency of 22 ms was evident [21]. SteamVR Tracking 1.0 further proved to be robust against partial and total occlusion [28].

Few studies [30,31] have yet investigated the current SteamVR Tracking 2.0 and the second generation SteamVR Base Stations 2.0. In these works, static position and orientation accuracy were analyzed [30,31]. The first study [30] involved a test facility for calibration of high-accuracy positioning instruments, showing that SteamVR Tracking 2.0 had high reproducibility of position measurement values in the millimeter range, yet transient magnitudes of several centimeters were observed. Tracking algorithm problems were reported as well for a scenario involving two base stations [30]. In the second study [31], SteamVR Tracking 2.0 was competitively compared against the Oculus Insight (Oculus Insight: https://ai.facebook.com/blog/powered-by-ai-oculus-insight/ (accessed on 20 December 2022)) tracking system of the Oculus Quest 2 (Oculus Quest 2: https://www.meta.com/de/quest/products/quest-2/ (accessed on 20 December 2022)).

**Table 1:** Related work overview stating used SteamVR Tracking version, number of base stations, GTS, analysis types, number of measurement runs, and occurrence of positional drifts.

Ref.	Year	Vers.	Base Stations	GTS	Analysis Type	Runs	Drifts
[4]	2019	1.0	2	Universal Robots UR5	SA: PA, OA	5 each	Yes
[21]	2017	1.0	2	2D grid	(1) SA: PA, OA with LOT (2) SA: PA, OA without LOT (3) SA: PA, OA with simulated LOT (4) TPA: SL	(1) 1 each (2) 1 each (3) 20, 10, and 20 (4) 6	No
[23]	2021	1.0	2	Comau NS-16-1.65	(1) SPA: PA, OA (2) SA: PA, OA (3) DA: PA, OA	(1) 10 each (2) 1 each (3) 1 each	No
[24]	2018	1.0	2	PhaseSpace	(1) SA: PA (2) SA: PA with PO (3) SA: PA without occlusion (4) SA: PA	(1) 5 (2) 20 (3) 1 (4) 2	Yes
[25]	2019	1.0	2	SCORBOT ER VII	SA: PA, OA	4 each	No
[29]	2019	1.0	2	Universal Robots UR5	DA: PA, OA	30 each	No
[26]	2018	1.0	2	Astrobee	(1) SA: PA, OA (2) DA: PA, OA	(1) 5 each (2) 4 and 3	No
[27]	2020	1.0	2	UKA LBR iiwa 14 R820	(1) DPA: PA, OA (2) DA: PA, OA	(1) 5 each (2) 1 each	No
[28]	2022	1.0	2	2D grid	(1) RA: intra- and intertracker accuracy (2) SA: PA with TO and PO	(1) 4 each (2) 4 each	Yes
[30]	2021	2.0	1, 2	Stable surveying pillars	(1) SPA: PA (2) SA: PA, OA	(1) 1 (2) 1 each	No
[31]	2021	2.0	4	2D grid	(1) RA: intertracker accuracy (2) SA: PA (3) SPA: jitter	(1) 1 (2) 1 (3) 1	No
This work		2.0	2, 4	Universal Robots UR10	(1) SA: PA, OA (2) DA: PA (3) SA: PA with PO	(1) 5 each (2) 5 (3) 5	No

Abbreviations for nomenclature used in this table: dynamic analysis—DA, dynamic precision analysis—DPA, loss of tracking—LOT, orientational accuracy—OA, partial occlusion—PO, positional accuracy—PA, reliability analysis—RA, static analysis—SA, static precision analysis—SPA, system latency—SL, time performance analysis—TPA, and total occlusion—TO.

## 3. Materials and Methods

In the following, materials and methods for evaluation of SteamVR Tracking 2.0 are elaborated. These comprise the description of used hardware and software components, experimental environment and setups, evaluation procedures, and the evaluation metric.

### 3.1. SteamVR Tracking Hardware and Software

SteamVR Tracking uses an inside-out principle [21] that involves hardware and software components. SteamVR Base Stations emit alternating horizontal and vertical infrared (IR) laser scans at a specific sweeping angle [4]. The surface of trackable hardware, e.g., a head mounted display, controllers, or a standalone tracker, is equipped with photodiodes to detect scans [21]. These determine Swept Angle Laser Tracking (SALT) information by themselves. Time differences between scan and perception allows position and orientation estimation [4]. While the first generation uses synchronized scans, the second generation uses a sync-on-beam signal to provide SALT information, making time-based calculation obsolete [25]. Furthermore, the first generation uses a sweeping angle of 120° at a sample rate of 60 Hz with two base stations, while the second generation uses 160° horizontal × 115° vertical at 100 Hz. Another novelty of the second generation is the possibility to use up to four base stations. While an area of 5×5 m can be covered using two base stations, four base stations extend this space up to 10×10 m. The base stations used in this work are four SteamVR Base Stations 2.0.

**Figure 1:**
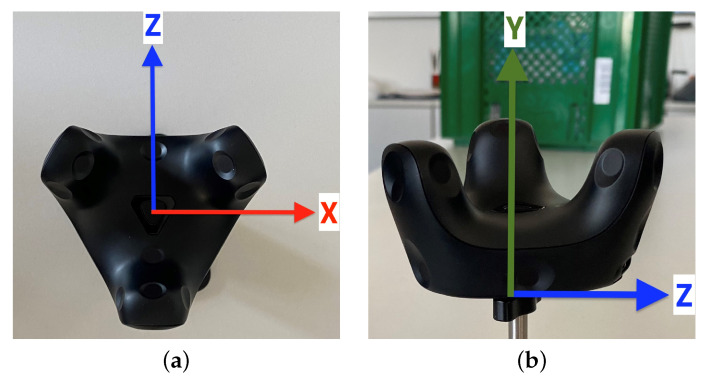
VIVE Tracker (3.0) with its coordinate system: (**a**) Top view with X- and Z-axis. (**b**) Side view with Y- and Z-axis.

For tracking accuracy evaluation, a VIVE Tracker (3.0) [32] is used, depicted in Figure 1, measuring 79.0×70.9×44.1 mm and weighing 75 g. Surface photodiodes allow a 240° field of view, and its right-handed coordinate system has its origin in the center of its bottom. The tracker is coupled with a host via a Universal Serial Bus (USB) 2.0 interface using a dongle, and its battery enables approx. 7.5 hours of operation. In addition to an ISO 1222:2010 [33]-compliant mount, an additional docking port enables to attach accessories.

In regards to the used software, the used hardware components demand the SteamVR Tracking 2.0 framework, for which Unity3D (Unity3D: https://unity3d.com/unity/whats-new/2021.1.25 (accessed on 18 February 2022)) in version 2021.1.25 serves as Application Programming Interface (API). The additional SteamVR plugin (SteamVR plugin: https://assetstore.unity.com/packages/tools/integration/steamvr-plugin-32647#description (accessed on 18 February 2022)) for Unity3D enables integration of SteamVR Tracking 2.0 to record tracker position and orientation. The SteamVR application (SteamVR application: https://store.steampowered.com/app/250820/SteamVR/ (accessed on 18 February 2022)) in version 1.20.4 is used to register involved hardware components and process received tracking data.

### 3.2. Universal Robots UR10 Robot Arm

The Universal Robots UR10 CB-Series robot arm is used to translate and rotate the VIVE Tracker (3.0) and serves as GTS, featuring a repeatability of ±0.1 mm according to ISO 9283:1998 [34]. Its construction comprises six rotating joints: base, shoulder, elbow, and three wrists. The tracker is mounted on the third wrist via an adapter with known dimensions. Each joint has a rotation range ±360°, allowing the robot arm to achieve a reach of 1300 mm at a maximum payload of 10 kg. The maximum velocity of base and shoulder joints is 120°/s, while that of all other joints is 180°/s. Resting on a foundation with a diameter of 190 mm, the whole construction weighs 28.9 kg, including power supply cables.

The Universal Robots Log Viewer (Universal Robots Log Viewer: https://www.universal-robots.com/download/software-e-series/support/ur-log-viewer/log-viewer-v1210/ (accessed on 18 February 2022)) in version 1.2.1.0 is used to collect positional and orientational data from the robot arm that serves as the ground truth for SteamVR Tracking 2.0 experiments, gathered with a sampling rate of 250 Hz.

**Figure 2:**
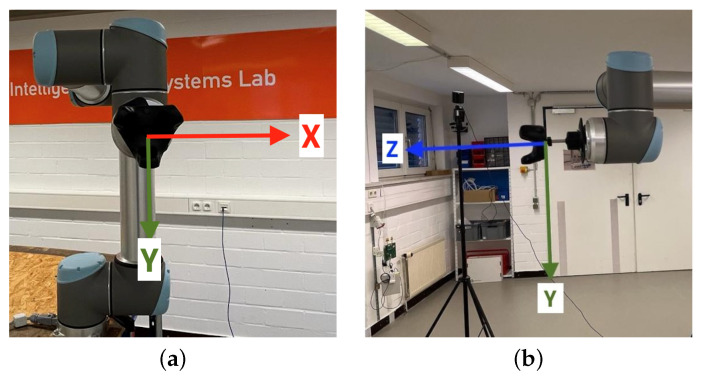
Universal Robots UR10 tool coordinate system with TCP adjusted to mounted VIVE Tracker (3.0) base center: (**a**) Frontal view with X- and Y-axis. (**b**) Side view with Y- and Z-axis.

Two predefined coordinate systems are available when operating the robot arm: the base coordinate system, with its origin in the center of the robot base, and the tool coordinate system, with its origin in a configurable Tool Center Point (TCP), originally located in the center of the third wrist. The latter one is relevant for experiments which is, like the coordinate system of the VIVE Tracker (3.0), a right-handed coordinate system. Based on the dimensions of the used adapter, the TCP was configured to be located at the center of the tracker base, i.e., the tracker coordinate system origin. The resulting build-up and robot arm coordinate system orientation is shown in Figure 2.

### 3.3. Experimental Environment and Setups

**Figure 3:**
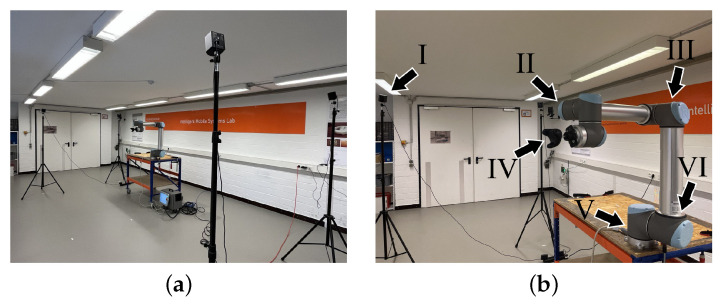
Experimental environment and setup: (**a**) overview of experimental environment and (**b**) details of experimental setup showing: (I) SteamVR Base Station 2.0, (II) Universal Robots UR10 robot arm wrist, (III) robot arm elbow, (IV) mounted VIVE Tracker (3.0), (V) robot arm base, and (VI) robot arm shoulder.

The environment experiments took place in was a robot laboratory with an area of 12.00 × 4.74 m. The room had two window fronts and, in addition to experiment setups, contained other equipment. This comprised, e.g., active electronic devices, such as lighting, computers, and routers, and reflective surfaces for robot guidance. Windows and reflective surfaces were intentionally left uncovered to serve as common and potentially challenging conditions in real-life working environments. An overview including an experiment setup is depicted in Figure 3a.

**Figure 4:**
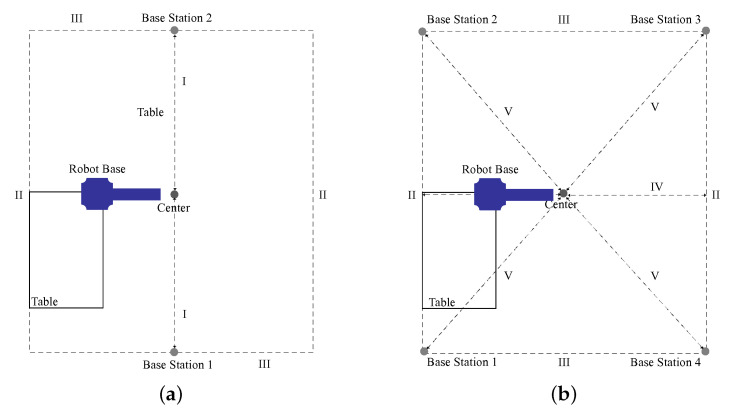
Experimental setup sketches, aerial view (ground level distances: (I) 2.68 m, (II) 5.36 m, (III) 2.00 m, (IV) 1.00 m, and (V) 2.86 m; heights: TCP/VIVE Tracker (3.0) 1.65 m and SteamVR Base Stations 2.0 2.10 m): (**a**) two-base-station setup and (**b**) four-base-station setup.

For comparison of the two and four SteamVR Base Stations 2.0, two experimental setups were realized, depicted as sketches in Figure 4. Both setups used the same area of 2.00 × 5.36 m. For the first setup, two base stations were placed in the short side centers of the setup area. For the second setup, four base stations were placed in the setup area corners. Base station distances to the setup area center were 2.68 m in the two-base-station setup and 2.86 m in the four-base-station setup. Base stations were positioned at a height of 2.10 m via tripods. On the left side of the setup area, a desk was positioned, with a working area measuring 1.20 × 0.80 m at a height of 0.80 m. The Universal Robots UR10 robot arm was mounted on the upper right corner of said desk, as shown in Figure 3b. The TCP/VIVE Tracker (3.0) was attached to the robot arm wrist via a rigid adapter at a precisely measured distance of 70 mm from the original TCP. It was located at a height of 1.65 m, measured from ground level. The tracker base center was configured as TCP, being the center of the setup with base stations aligned towards it.

### 3.4. Evaluation Procedures

Procedures for the evaluation of SteamVR Tracking 2.0 performance comprise an analysis for static translation and rotation accuracy, an analysis for dynamic translation accuracy, and an analysis for static translation occlusion robustness. In the following, these are referred to as “static analysis”, “dynamic analysis”, and “occlusion analysis”. Each analysis was performed for both experiment setups with, respectively, two and four SteamVR Base Stations 2.0, except the occlusion analysis directly focusing on four base stations. At experiment starts, as well as at each setup, switch SteamVR Tracking was calibrated according to the SteamVR protocol. The following paragraphs describe details of the focus and conducted evaluation procedures of each of the tree analyses.

The static analysis focused on tracking performance when both the object to be tracked and the tracking system are static to each other, i.e., start and end points, but no motion is evaluated. Here, the outcome of two independent aspects, translation and rotation, were evaluated. Five measurement runs with 300 single measurements for accuracy determination, i.e., 100 for each axis, were conducted. Translations/rotations were performed with a velocity of 2.5 mm/s. For the translational accuracy measurements, the TCP of the robot arm was translated from the start point $p_{0}$ to end point p±100.0 mm on each axis, X, Y, and Z. For rotational accuracy measurements, the TCP was rotated around the X- and the Y-axis from the starting point $p_{0}$ to end point p±30° and around the Z-axis to end point p±60°. This did not involve any translation of the TCP, hence it remained on its initial position in space. The extended rotation range of ±60° around the Z-axis was chosen to ensure comparability to prior work [4] that chose this range based on requirements for ultrasound simulation. Measurements for end point *p* were taken 10 s after arrival of the robot arm in its designated resting position. This was performed to avoid any oscillations potentially caused by momentum.

**Figure 5:**
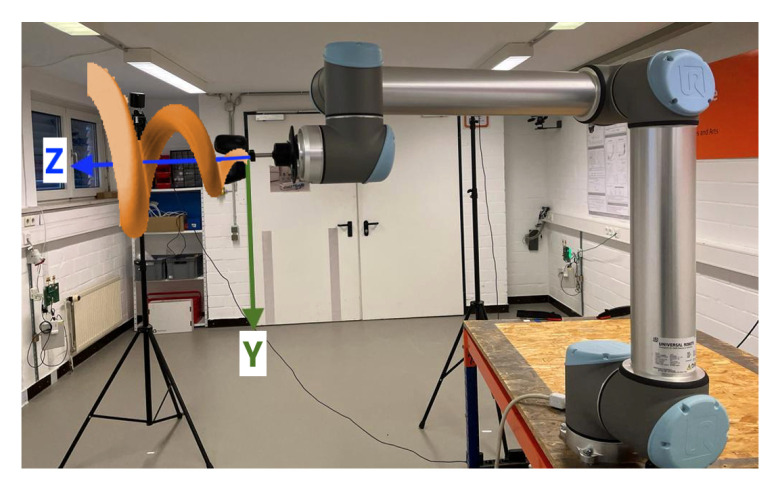
Side view of spiral trajectory around the Universal Robots UR10 robot arm TCP Z-axis.

The dynamic analysis focused on tracking performance while the object being tracked is in motion. Five measurement runs were carried out using three different translation velocities: 12.5 mm/s, 25 mm/s, and 50 mm/s, referred to as “slow”, “medium”, and “fast” in the following. Due to the static sample rates, 3858 and 2740, respectively, 1611 single measurements per run were gathered during experiments for the three velocities. The TCP performed a conical spiral motion, moving along the Z-axis with an expanding circulation radius over the X- and Y-axis, as defined via Equations (1)–(3), with r(φ) being the archimedean spiral and *s* being the slope on the Z-axis. Its actual movement in space is further delineated in Figure 5. The specific sequence implementation by the robot arm can be comprehended via support documents (Universal Robots Support: https://www.universal-robots.com/articles/ur/programming/circular-path-using-movepmovec/ (accessed on 20 December 2022)). The unrolled spiral had a length of 200 mm.

(1) $$\begin{array}{cc} x= & \;r(\varphi )\times \cos(\varphi ) \end{array}$$

(2) $$\begin{array}{cc} y= & \;r(\varphi )\times \sin(\varphi ) \end{array}$$

(3) $$\begin{array}{cc} z= & \;z_{0}+s\times r\left(\varphi \right) \end{array}$$

The occlusions analysis focused on tracking performance when the object to be tracked experienced a partial occlusion of a base station. Four different occlusion experiments with five measurement runs each were conducted, related to the those performed in the static analysis and using the same translation sequence. In each experiment, one distinct base station was hidden from the tracker via occlusion in the order of the base station numbering shown in Figure 4b, i.e., in the first experiment base station 1 was hidden, in the second experiment base station 2 was hidden, etc.

Sampling rates and coordinate systems of SteamVR Tracking and the robot arm were harmonized to achieve a reliable parameter determination and measurement result comparison. This was conducted via post-processing after data acquisition. The 250 Hz sampling rate of the robot arm was downsampled to the 100 Hz rate of SteamVR Tracking 2.0. In a filter operation, the mean of surrounding values were determined with an appropriate window size of 5. The robot arm coordinate system was adapted to that of SteamVR Tracking. For this purpose, a transformation matrix was determined for each axis component and combined into an overall displacement matrix. Translations and mirroring were required for transformation of experiment series.

### 3.5. Evaluation Metric

The Root Mean Square Error (RMSE) was chosen to evaluate the measurement results to align with the majority of related work [4,21,23,24,25,27]. It is considered as deviation between observed values and values determined by a model. In the context of experiments, SteamVR Tracking is put into relation with the robot arm GTS. Hence, the RMSE determines the deviation between SteamVR Tracking estimations for the tracker and the actual robot arm ground truth for its TCP. The RMSE is given by Equation (4), where *N* is the number of samples and $\left(p_{r_{i}}-p_{t_{i}}\right)$ is the difference between the robot arm position data ($p_{r}$) and that of SteamVR Tracking ($p_{t}$).

(4) $$\mathrm{RMSE}=\sqrt{\frac{1}{N}\sum_{i=1}^{N}{\left(p_{r_{i}}-p_{t_{i}}\right)}^{2}}$$

For each axis component X, Y, and Z, the RMSE is calculated for the direction of movement. Furthermore, to determine tracking errors in all directions, the average error for all axis components is calculated. Both calculations are referred to as “movement direction” and “mean in all directions” in the following. In addition to the RMSE for individual axis components, the aggregated RMSE for both over all axes is calculated as well. Results reported in the following represent the mean RMSEs of the five measurement runs conducted for each analysis, reported together with their standard deviation.

## 4. Results

The following subsections report results of the conducted experiments for the static analysis, the dynamic analysis, and the occlusion analysis with, respectively, two and four SteamVR Base Stations 2.0.

### 4.1. Static Translation and Rotation Accuracy

Table 2 presents results of the translation experiments of the static analysis. In general, both setups achieved a high translational tracking accuracy with errors in the submillimeter range. However, in the two-base-station setup, these were notably higher in comparison with those in the four-base-station setup. This applies for the movement direction, as well as for the mean in all directions. While errors in the two-base-station setup lie within the mid-submillimeter range, those in the four-base-station setup lie within the low-submillimeter range. Standard deviations also indicate an increased error scattering for the two-base-station setup, while those for the four-base-station setup are notably low.

Table 3 presents results of the rotation experiments of the static analysis. Similar to outcomes of translation experiments, both setups achieved a high rotational tracking accuracy with error in the subdegree range. Again, increased errors in both the movement direction and the mean in all directions were measured in the two-base-station setup. While errors in the two-base-station setup usually lie within the mid-subdegree range, those in the four-base-station setup usually lie within the low-subdegree range. However, there are several deviations. For the movement direction of the two-base-station setup, the X-axis error is notably high, and that of the Z-axis notably low within the subdegree range. Standard deviations indicate a low error scattering for both setups. In addition, results for the four-base-station setup show an increased error in the direction of the X-axis due to a single outlier. Excluding it results in notably lowered errors and standard deviations for the movement direction X-axis, as well as over all axes. This accounts for the mean in all directions as well.

### 4.2. Dynamic Translation Accuracy

Table 4 presents the results of the translation experiments of the dynamic analysis. In general, experiments conducted with the four-base-station setup show considerably lower tracking errors over all velocities compared with those using two base stations. A notably high accuracy in the mid-to-upper submillimeter range was achieved at a slow velocity using four base stations. Errors over all axes measured with the two-base-station setup are increased many times over those of the four-base-station setup, independent of the used velocity. Scattering visible in the standard deviations measured for both setups is considerably lower for all velocities in the four-base-station setup. In comparison with the four-base-station setup, the two-base-station setup showed higher and often notably increased errors for the X- and Y-axis over all velocities. Those for the Y-axis increased moderately with increasing velocity. In the four-base-station setup, a comparable but less pronounced phenomenon was observed only for the fast velocity.

**Figure 6:**
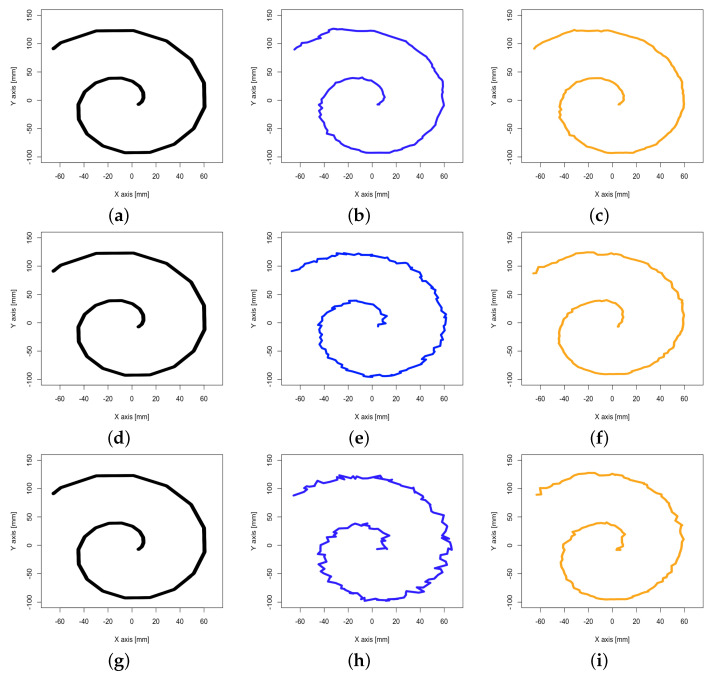
Mean spiral trajectory at velocities 12.5 mm/s (slow), 25 mm/s (medium), and 50 mm/s (fast) for Universal Robots UR10 robot arm (black) with mounted VIVE Tracker (3.0) and setups with two (blue) and four (orange) SteamVR Base Stations 2.0: (**a**) Robot arm, slow. (**b**) Two base stations, slow. (**c**) Four base stations, slow. (**d**) Robot arm, medium. (**e**) Two base stations, medium. (**f**) Four base stations, medium. (**g**) Robot arm, fast. (**h**) Two base stations, fast. (**i**) Four base stations, fast.

Figure 6 visualizes the corresponding spiral trajectories performed by the Universal Robots UR10 robot arm TCP and positions measured by the Vive Tracker (3.0) as mean over all runs. Outliers and irregularities are visible for all velocities in both setups. Their magnitude positively correlates with increased velocity. However, these are notably less pronounced in the four-base-station setup.

### 4.3. Static Translation Robustness

Table 5 presents the results of the occlusion analysis. Independent from the occluded SteamVR Base Station 2.0, a low error within the submillimeter range was observed in all experiments. Moderate deviations are ascribed to outliers in single measurement runs, which occurred during occlusion of base station 1 and base station 3. On the X-axis, errors for the movement direction are notably lower than those for other axes. As there are no noteworthy deviations between distinct occlusion experiment outcomes, no specific base station can be assumed to have excess impact on the track accuracy. However, in comparison with results of the translation experiments during the static analysis, reported in Table 2, partial occlusion results in noteworthy higher errors. These are also higher than those measured with the two-base-station setup.

## 5. Discussion

The following sections discuss findings, providing interpretations for different experiment results and addressing factual as well as potential limitations.

### 5.1. Static Tracking Accuracy

Both SteamVR Tracking 2.0 setups with two and four SteamVR Base Stations 2.0 achieve a high translational and rotational static tracking accuracy with an error below 1 mm, respectively, 1 ° in a nonlaboratory environment. The use of four base stations notably increases translational accuracy, effectively doubling it by decreasing scattering. Positive effects are even more pronounced for the rotational accuracy, which can be increased by many times. However, the occurrence of outliers can lead to accuracy drops, as seen in one rotation measurement run. This may of course apply for translational accuracy as well. Nevertheless, the use of four base stations provides a considerably better overall performance in comparison with that achieved with two base stations.

The achieved static analysis results for two base stations are comparable to that of SteamVR Tracking 1.0 [21,23]. In comparison with prior work [4] using SteamVR Tracking 1.0 as well, more accurate results are observed for rotations. In a direct comparison with the research using SteamVR Tracking 2.0 [30,31], considerably better results in overall static tracking accuracy are observed. The tracking algorithm issues when using four base stations reported in these works played only a minor role in this work. Nevertheless, four base stations enabled a generally higher accuracy. Comparable results in the submilimeter and subdegree were shown by other related research [27,29] in tracking accuracy via static analyses.

Findings of the static analysis grant detailed insights into the translational and rotational baseline accuracies of SteamVR Tracking 2.0. These are considered high, indicating the best potentially achievable accuracies for dynamic analyses.

### 5.2. Dynamic Tracking Accuracy

The SteamVR Tracking 2.0 setup using four SteamVR Base Stations 2.0 achieved a generally higher dynamic translation accuracy than that using two base stations, with an error below 1 mm at a slow velocity. Even though an increased velocity generally resulted in an impaired tracking accuracy in both setups, that in the four-base-station setup only decreased moderately. Notably higher errors for increased velocities observed in the two-base-station setup suggest tracking issues related to fewer information from base station scans. This assumption may also be supported by considerably larger deviations of axis-related accuracies of those measured for the X- and the Z-axis, not observed in the four-base-station setup. However, as setups differ in regards to base stations positioning, observations may also be related to this aspect.

In addition, other issues seem to be related to the use of solely two base stations. Other studies using two base stations with SteamVR Tracking 1.0 report temporary drops in measurement frequency, as well as disconnections [4,21]. During experiments conducted in this work, no such events were observed; this might indicate an increased system stability of SteamVR Tracking 2.0. Occlusions by GTSs, such as a robot arm from a base station’s point of view, may also be responsible axis-specific decreases in dynamic translation accuracy [29]. Yet, such obstacles can be ruled out for the two-base-station setup used in this work. Dynamic translation accuracy losses for increased velocities were also reported for SteamVR Tracking 1.0 [23], yet these are not comparable with those observed in this work due to considerable differences in used velocities.

Findings of the dynamic analysis indicate appropriateness of SteamVR Tracking 2.0 for a broad set of various medical use cases when using four base stations. Achieved translational accuracy for different velocities during challenging spiral trajectories is assumed to be decent enough for computer-aided rehabilitation practices and medical device tracking in VR-based training simulations.

### 5.3. Occlusion Robustness

Results of the occlusion analysis experiments with the four-base-station setup show a high translational static tracking accuracy during occlusion of single base stations, with errors below 1 mm. These indicate a high robustness against information loss from single base stations. However, it should be noted that errors are considerably higher than those measured for the two-base-station setup during static analysis translation experiments. This indicates that occlusion may actually be a challenging issue for SteamVR Tracking 2.0 in a four-base-station setup, even though three remaining base stations provide information via scans. Outliers, as those experienced during single measurement runs, can impair the tracking error to leave the submillimeter range. Errors are assumed to further increase in the case of full occlusion of more than one base station. This suggests the importance of base station positioning in challenging experiment environments with vast occlusion potential. Well-designed setups using two base stations with no risk of full occlusion may thus outperform ones with four base stations that bear such risks.

As no partial occlusion analysis has yet been performed for SteamVR Tracking 2.0, no direct comparison with related work is possible.

Findings of the occlusion analysis indicate a robustness appropriate for different medical use cases involving challenging environments and scenarios, e.g., simulated operation theaters with moving people or physiotherapy practices in which body parts and positioning may occlude trackers.

### 5.4. Limitations

Both translucent light from two window fronts and the reflections from reflective surfaces could have had an effect on measurements. This may also apply for radio signals from other devices within the experiment environment. Even though the Universal Robots UR10 robot arm was evaluated via KC Registration (KC Registration: https://www.korea-certification.com/kc/ (accessed on 20 December 2022)) for use in working environments, a theoretical risk of interference remains. Furthermore, potential temporary occlusions of the Vive Tracker (3.0) by the robot arm during movements may have affected SteamVR Base Station 2.0 scans. In addition, the table on which the robot arm base was mounted was not bolted to the floor, which might have caused mild shifts during movements due to to weight displacement.

Even though measures for risk minimization of the undesired movement of setup components were taken, mild vibrations of the building due to moving people, traffic, or wind cannot be ruled out. These may have had an influence on measurement outcomes within the submillimeter and subdegree range. Furthermore, an influence of radio sources outside of the experiment environment cannot be ruled out either. Another aspect that might have affected the measured tracking performance is approximation errors from harmonization methods applied for both sampling rates and coordinate systems of the robot arm and SteamVR Tracking 2.0.

In addition, not all relevant aspects of SteamVR Tracking 2.0 could have been evaluated by the presented research, as their extent was outside the scope of its project-bound context. These comprises a dynamic analysis for rotation accuracy and an occlusion analysis for multiple partial occlusions of base stations. Analysis combinations, e.g., a dynamic analysis involving occlusions, may be of interest as well.

## 6. Conclusions

SteamVR Tracking 2.0 achieves high static translation and rotation tracking accuracy with two and four SteamVR Base Stations 2.0 in the submillimeter and subdegree range. Yet, four base stations enable considerably higher accuracy with less scattering. This also applies for dynamic translation accuracy, in which submillimeter accuracy of translations could only be achieved via four base stations at a slow velocity. Translational accuracy suffers from increasing velocity, especially in the two-base-station setup. Here, the limited information during dynamic translation can also result in axis-specific accuracy deterioration, potentially related to base station positioning as well. Four base stations enable generally robust accuracy during partial occlusion of single base stations. This, however, appears to stress the tracking algorithm considerably, resulting in a lower accuracy than measured with two base stations.

Setups involving four base stations are assumed to be more suitable for use cases in which occlusions may occur. However, appropriateness of SteamVR Tracking 2.0 in scenarios demanding a high dynamic tracking accuracy during fast motions of tracked objects may be limited.

Future work on the topic will focus on evaluation of SteamVR Tracking 2.0 in regard to dynamic analyses and additional occlusion scenarios that have not yet been addressed. Exploring alternative setups, orientations, and distances is of interest as well. SteamVR Tracking 2.0 will further be evaluated for the use case of computer-aided physiotherapy practice in combination with VR-based simulations.

## Figures and Tables

**Table 2:** Errors (mm) in tracking of translation with standard deviations (sd), five measurement runs for each movement, and the best results are highlighted.

RMSE	Axis	Two Base Stations mm (*sd*)	Four Base Stations mm (*sd*)
Movement direction	X	0.393 (0.363)	**0.085** (0.083)
	Y	0.345 (0.347)	**0.199** (0.117)
	Z	0.551 (0.499)	**0.158** (0.112)
	All	0.429 (0.403)	**0.155** (0.104)
Mean in all directions	X	0.296 (0.455)	**0.167** (0.107)
	Y	0.458 (0.418)	**0.223** (0.096)
	Z	0.386 (0.335)	**0.101** (0.074)
	All	0.435 (0.217)	**0.171** (0.092)

**Table 3:** Errors (°) in tracking of rotation with standard deviations (sd), five measurement runs for each movement, and the best results are highlighted.

RMSE	Axis	Two Base Stations ° (*sd*)	Four Base Stations ° (*sd*)
Movement direction	X	0.827 (0.029)	**0.375**^a^ (0.297) ^a^
	Y	0.474 (0.126)	**0.086** (0.066)
	Z	0.125 (0.053)	**0.084** (0.076)
	All	0.574 (0.069)	**0.227**^a^ (0.146) ^a^
Mean in all directions	X	0.493 (0.065)	**0.234**^a^ (0.143) ^a^
	Y	0.385 (0.160)	**0.222**^a^ (0.142) ^a^
	Z	0.503 (0.046)	**0.203**^a^ (0.131) ^a^
	All	0.460 (0.090)	**0.220**^a^ (0.139) ^a^

^a^ Caused by outlier in the fifth run. When excluded, RMSEs for movement directions are: 0.086° (0.021) for X and 0.085° (0.054) for all; RMSEs for mean in all directions are: 0.091° (0.045) for X, 0.082° (0.058) for Y, 0.086° (0.081) for Z, and 0.086° (0.061) for all.

**Table 4:** Errors (mm) in tracking of translation with standard deviations (sd) during the conical spiral movement with different velocities: 12.5 mm/s (slow), 25 mm/s (medium), and 50 mm/s (fast). The best results are highlighted.

RMSE	Axis	Two Base Stations			Four Base Stations		
		Slow mm (*sd*)	Medium mm (*sd*)	Fast mm (*sd*)	Slow mm (*sd*)	Medium mm (*sd*)	Fast mm (*sd*)
Movement direction	X	6.130 (5.872)	11.320 (9.215)	14.377 (15.217)	**0.316** (0.444)	2.020 (1.998)	5.537 (5.421)
	Y	0.899 (0.912)	1.990 (2.146)	4.727 (5.231)	**0.637** (0.513)	1.994 (1.876)	2.144 (1.965)
	Z	1.165 (1.231)	13.910 (12.731)	15.432 (15.144)	**0.823** (0.781)	2.508 (2.322)	4.376 (4.451)
	All	2.824 (2.672)	8.664 (8.031)	11.091 (11.864)	**0.574** (0.579)	2.164 (2.065)	4.237 (3.946)

**Table 5:** Errors (mm) in tracking of translation with standard deviations (sd) of experiments with occluded SteamVR Base Stations 2.0; the numbering corresponds to that of Figure 4b.

RMSE	Axis	Base Station Occlusion			
		Base Station 1 mm (*sd*)	Base Station 2 mm (*sd*)	Base Station 3 mm (*sd*)	Base Station 4 mm (*sd*)
Movement direction	X	0.108 (0.102)	0.095 (0.089)	0.242 (0.232)	0.142 (0.121)
	Y	0.636 (0.545)	0.637 (0.589)	1.318 ^b^ (0.914) ^b^	0.861 (0.774)
	Z	1.663 ^a^ (1.487) ^a^	0.777 (0.654)	0.930 (0.887)	0.840 (0.762)
	All	0.781 ^a^ (0.711) ^a^	0.531 (0.444)	0.823 ^b^ (0.678) ^b^	0.583 (0.552)
Mean in all directions	X	0.605 (0.547)	0.345 (0.421)	0.638 (0.429)	0.441 (0.532)
	Y	0.518 (0.465)	0.699 (0.327)	0.974 ^b^ (0.678) ^b^	0.689 (0.546)
	Z	1.093 ^a^ (0.568) ^a^	0.789 (0.487)	0.989 (0.478)	0.661 (0.525)
	All	0.738 ^a^ (0.699) ^a^	0.611 (0.528)	0.867 ^b^ (0.528) ^b^	0.597 (0.534)

^a^ Caused by outlier in the third run. When excluded, RMSEs for movement directions are: 0.703° (0.748) for Z and 0.567° (0.465) for all; RMSEs for mean in all directions are: 0.544° (0.562) for Z and 0.534° (0.524) for all. ^b^ Caused by outlier in the fifth run. When excluded, RMSEs for movement directions are: 0.804° (0.728) for Y and 0.633° (0.615) for all; RMSEs for mean in all directions are: 0.611° (0.348) for Y and 0.704° (0.418) for all.

## Data Availability

Data gathered during experiments are available by request to the corresponding author.

## Abbreviations

The following abbreviations are used in this manuscript:

API	Application Programming Interface
GTS	Ground Truth System
IR	Infrared
RMSE	Root Mean Square Error
TCP	Tool Center Point
USB	Universal Serial Bus
VR	Virtual Reality
